# Earliest Mechanisms of Dopaminergic Neurons Sufferance in a Novel Slow Progressing Ex Vivo Model of Parkinson Disease in Rat Organotypic Cultures of Substantia Nigra

**DOI:** 10.3390/ijms20092224

**Published:** 2019-05-06

**Authors:** Matteo Dal Ben, Rosario Bongiovanni, Simone Tuniz, Emanuela Fioriti, Claudio Tiribelli, Rita Moretti, Silvia Gazzin

**Affiliations:** 1 Department of Medical, Surgical, and Health Sciences, University of Trieste, 34100 Trieste, Italy; mdalben@fegato.it; 2 Fondazione Italiana Fegato, AREA Science Park, 34149 Trieste, Italy; rbongiovanni@fegato.it (R.B.); simone.tuniz@fegato.it (S.T.); emanuela.fioriti@fegato.it (E.F.); ctliver@fegato.it (C.T.); 3 Neurology Clinic, Department of Medical, Surgical, and Health Sciences, University of Trieste, 34100 Trieste, Italy; moretti@units.it

**Keywords:** brain organotypic cultures, dopaminergic neurons, causative mechanisms, Real-Time PCR, glutamate neurotoxicity, dopamine, neurodegeneration, neuron morphometry

## Abstract

The current treatments of Parkinson disease (PD) are ineffective mainly due to the poor understanding of the early events causing the decline of dopaminergic neurons (DOPAn). To overcome this problem, slow progressively degenerating models of PD allowing the study of the pre-clinical phase are crucial. We recreated in a short ex vivo time scale (96 h) all the features of human PD (needing dozens of years) by challenging organotypic culture of rat substantia nigra with low doses of rotenone. Thus, taking advantage of the existent knowledge, the model was used to perform a time-dependent comparative study of the principal possible causative molecular mechanisms undergoing DOPAn demise. Alteration in the redox state and inflammation started at 3 h, preceding the reduction in DOPAn number (pre-diagnosis phase). The number of DOPAn declined to levels compatible with diagnosis only at 12 h. The decline was accompanied by a persistent inflammation and redox imbalance. Significant microglia activation, apoptosis, a reduction in dopamine vesicle transporters, and the ubiquitination of misfolded protein clearance pathways were late (96 h, consequential) events. The work suggests inflammation and redox imbalance as simultaneous early mechanisms undergoing DOPAn sufferance, to be targeted for a causative treatment aimed to stop/delay PD.

## 1. Introduction

Parkinson disease (PD) is the second most common neurodegenerative disorder after Alzheimer’s disease [1]. The core pathologic feature of motor PD is the reduced production of the neurotransmitter dopamine in dopaminergic neurons (DOPAn) in the substantia nigra pars compacta (SN). The main consequence is the alteration of the nigrostriatal pathway, causing motor imbalance, associated to the recently acquired alterations of the other three dopaminergic pathways (the mesolimbic, the mesocortical and the tuberoinfundibular system) involved in the altered non-motor functions, constantly described in PD [2,3,4,5,6].

Nowadays is clear that PD is a multifactorial pathology. Several genes have been identified as risk-factors, but very few cases (about of 5–10%) are related to genetic mutations, and the majority of Parkinson cases are classified as sporadic [7,8]. Older age and male sex are a risk factor in developing PD [9]. Environmental factors, occupational exposure to heavy metals, as well specific pesticides are also responsible for the pathogenesis [7].

From a molecular point of view, an incredible amount of work has been done in the past decades in every available experimental model (in silico, in vitro, ex vivo, in vivo, toxin induced, and genetically modified) to unravel the molecular determinants of dopamine demise. Demonstrated components are oxidative stress, inflammation, microgliosis, excitotoxicity, apoptosis, autophagy, mitochondrial dysfunction, alterations in the endosomal-lysosomal functioning, vesicle trafficking, and the ubiquitination mediated clearance of unfolded protein, until the most recent involvement of epigenetics [3,8,10,11,12,13,14,15,16,17,18].

Nevertheless, the etiology of PD is still elusive [5,8]: a comparative, time-course evaluation able to unravel the sequence of the molecular events of the very early, pre-clinical phase DOPAn sufferance, is still not available. This might be the reason why we still do not have curative therapies but just a temporary control for symptoms [12,17,19,20].

To unravel the onset of DOPAn demise, a dynamic (time-course) slowly progressing approach comparing multiple potential causative mechanisms is crucial [21]. This is unlikely given that the vast majority of the old and most recent literature preferred an end-point experimental set-up, classically representative of an already evident pathology, or coming from the autopsy. Indeed, the vast majority of the published studies investigated only specific processes (e.g., only inflammation, only redox, only growth factors) avoiding an integrative vision of the degenerative disorder and preventing a proper description of the sequence of events from the onset, up to the final stages of PD progression. One original work tried to compare the higher number of potential causative mechanisms by a time-course approach [22] but, due to the acute scheme (see later into discussion), all the investigated genes were already activated at the earliest time (3 h).

For this reason, we decided to address the time course of the molecular events ongoing to DOPAn demise by developing an ex vivo chronic (96 h) model of PD characterized by a slow and progressive degeneration of dopaminergic cells of the SN, reproducing all the features of the human pathology (needing dozen of years) [23]. The primary relevance of the model was that we gained access the pre-diagnostic phase, addressing the earliest (potentially causative) molecular mechanisms ongoing with the very first dopaminergic neurons sufferance. 

## 2. Results

### 2.1. Model Development and Experimental Plan

To gain access to the early, causative molecular mechanisms undergoing DOPAn sufferance, possibly preceding diagnosis, it was crucial to set-up a slow progressive PD model. 

To reproduce all the features of human pathology in an in vitro time scale (96 h), we combined the rat brain organotypic cultures of substantia nigra, maintaining in vitro the architecture, cellular heterogeneity, and metabolic features of the in vivo tissue, with low dose of rotenone. Rotenone is a pesticide known to induce PD in human [24,25,26], and reproducing in rats all of the behavioral, morphological and molecular features reminiscent of human PD [27,28]. 

**Figure 1:**
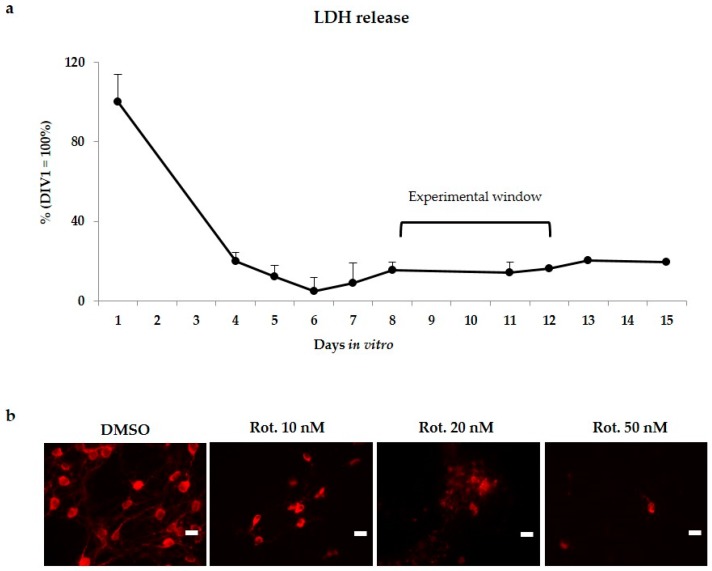
Experimental plan set-up. (**a**) LDH release in unchallenged cultures (indicative of culture recovery from the slicing procedure), and chose experimental window for rotenone challenging; (**b**) Representative immunofluorescences of DOPAn appearance evaluated by tyrosine hydroxylase (TH+) immunofluorescence staining (red signal) under 50, 20 and 10 nM rotenone (Rot.) challenging for 96 h. Scare bar 25 μm.

Because the cutting procedure to generate brain slices determines an important tissue sufferance, as first we followed the LDH release (lactate dehydrogenase, a marker of membrane leakage) during the first days in culture, without any challenging. Because challenging already suffering slices may introduce bias in the model, we waited the normalization of LDH (indicative of slices recovery). As shown in Figure 1a, after a relevant LDH release in the first 2–3 days, the LDH level stabilized at around 7 days, remaining constant at least for 15 days in vitro. Based on this information, we started the challenging at 8 days in vitro. Notably, the experiment (96 h long at maximum) finished largely in the plateau of LDH release.

The concentration of rotenone required to develop a slow progressive demise of DOPAn was experimentally determined. After recovery from the stress due to the slicing procedure Figure 1a, the cultures were exposed to the chemical. Three decreasing (from 50 to 10 nM) concentration were tested, and the appearance of DOPAn was evaluated by tyrosine hydroxylase (TH+) immunofluorescence staining (Figure 1b) at the end of the trial (96 h). As Figure 1b shows, 50 nM of rotenone induced the complete disappearance of DOPAn, few remnants were observable at 20 nM of rotenone, while 10 nM of the pesticide induced only a partial DOPAn TH+ signal loss. This concentration was applied thereafter in the study.

### 2.2. Morphometric Analysis of the Temporal Degeneration of Dopaminergic Neurons and Agreement with the Clinical Scenario

It has been established that most of the diagnoses occur when the degenerative process is advanced, with an estimated death of 40–60% of DOPAn, leading to the loss of 80% of putamen dopamine content [3,11,29,30]. To identify the different steps of the morphological degeneration of dopaminergic neurons under low (10 nM) concentration of rotenone, we assessed the number of TH+ DOPAn, the number of dopaminergic neuron’s neurites, as well as their length at 3, 12, 24, 48 and 96 h of rotenone challenging.

#### 2.2.1. Decrease of the Number of the DOPAn’s Neurites

**Figure 2:**
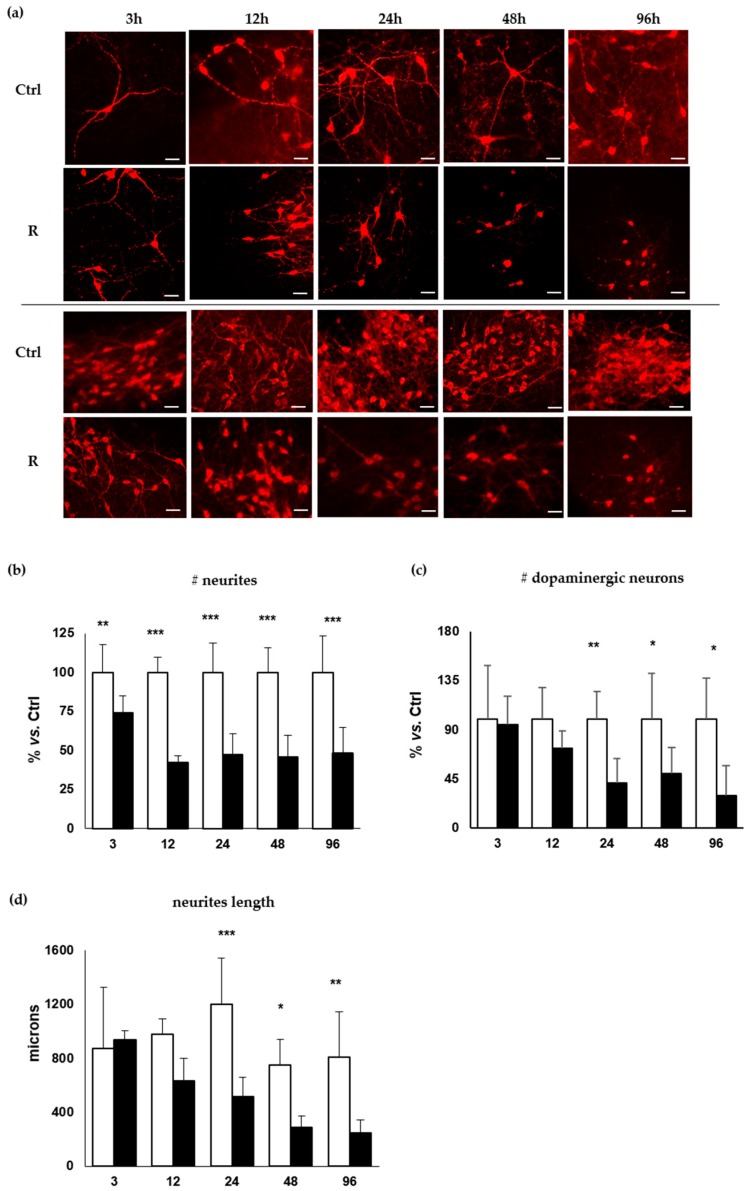
Analysis of the temporal degeneration of dopaminergic neurons. DOPAn appearance evaluated by tyrosine hydroxylase (TH+) immunofluorescence staining (red signal). Ctrl: controls. R: rotenone challenging. (**a**) Representation of DOPAn neurites changes (Upper two lines: both Ctrl and R, scale bar: 50 µm) and DOPAn number decrease (Lower two lines: both Ctrl and R, scale bar 100 µm); (**b**–**d**) Analysis of DOPAn demise; (**b**) Number of neurites; (**c**) Number of dopaminergic neurons. (**d**) Length of neurites (μm). White bars: DMSO. Black bars: Rotenone challenged slices. *x* axis: hours. Data are expressed as the mean ± S.D. of at least five biological repetitions. Statistical significance: * *p* < 0.05; ** *p* < 0.01; *** *p* < 0.001.

The first sign of DOPAn sufferance was the reduction of the number of neurites (Figure 2b), dropping after 3 h by 26% (*p* < 0.01) in challenged slices. This decrease is considered not sufficient to lead to symptom [29], thus it is representative of a pre-diagnosis clinical scenario. Later on (12, 24, 48, 96 h, all *p* < 0.001), the reduction of the number of neurites stabilizes to values of about of 50–60% of controls. The results at 12–96 h agree with Cheng [29], estimating a 50–60% axon terminal loss the time of the first diagnosis of PD [29]. Moreover, this finding agrees with the recent clinical observation of a retrograde axis in the morphological changes involving DOPAn, beginning in the distal neuritis and proceeding towards (in the direction of) the cell bodies, as recently observed by a PET (positron emission tomography) scan in early PD patients with a Hohen & Yhar score (HY) [31,32] of 1–2 [33]. No changes in the number of DOPAn neurites were detected in control OBCs during the experimental period.

#### 2.2.2. Decrease of the Number of the DOPAn

The number of TH+ DOPAn was not significantly affected at the early times (3–12 h, Figure 2c), reaching a maximal reduction of 27% at 12 h. At 24, 48, 96 h the decrease was more relevant, reaching the statistical significance (60%, 50%, 70%; *p* < 0.01, *p* < 0.05, *p* < 0.05, respectively). This picture well reproduces the human pathology as described by the Braak’s staging [34] and HY scale [31]. Specifically, motor symptoms appear at HY stage 1–2 [31] and Braak’s stage 2–3 [29,33,35], when the 30% of DOPAn TH+ and 60% of dopamine are lost (29); timing corresponding to the 12 h in our model. Thereafter DOPAn TH+ loss progresses up to 50% at Braak’s stage 4, corresponding to 24–48 h in our model, till to 60–70% DOPAn TH+ loss at the Braak’s stage 6 [23,29,33,34,35,36], corresponding to 96 h in our model. No changes in the number of DOPAn have been detected in control OBCs during the experimental period.

#### 2.2.3. Decrease of the Length of the DOPAn’s Neurites

Similarly, the neurite length (Figure 2d) started declining at 24 h (35%), becoming significantly reduced at 24, 48 and 96 h (60%, 60% and 70%; *p* < 0.001, *p* < 0.05 and *p* < 0.01, respectively). No changes in the length of neurites were detected in control OBCs all long the experimental period.

Altogether, the morphological analysis confirms we reached the goal of creating a slow progressing ex vivo system, supporting its use to represent the human pathology and allowing the analysis of the early (possibly causative) mechanisms ongoing DOPAn sufferance. As it is, the 3 h experimental time will offer the opportunity to focus on an early temporal window (pre-diagnosis) actually not accessible in a clinic. 

### 2.3. Time-Dependent Comparative Evaluation of the Molecular Events Undergoing DOPAn Sufferance and Demise

To identify the earliest (causative) molecular events undergoing DOPAn sufferance and demise, we performed a time-dependent comparative study of selected markers for the principal possible mechanisms underlining DOPAn degeneration and PD (Table 1). To this end, we selected markers already validated (see below).

#### 2.3.1. Real-Time PCR Analysis of Inflammation, Redox Imbalance, Unfolded Proteins Ubiquitination, Vesicles Transport, and Apoptosis; and Quantification of Glutamate in the Medium

Redox stress has been mentioned as the first-hint in PD development [6,37]. To monitor redox imbalance, we chose Heme-oxygenase 1 (*Hmox1*), noticed up-regulated in PD patients and considered a redox sensor [38,39,40,41], and sulfiredoxin1 (*Srnx1*), an adaptive response to redox stress, potentially protecting DOPAn from ROS toxicity [38,42,43,44]. 

Inflammation is a recognized component of PD. A heterogeneous panel of inflammatory molecules have been reported in literature, possibly representing different stages of the inflammatory cascade activated in the numerous models, as well during the progression of the diseases in patients. For this reason we chose multiple markers: Tumor necrosis factor alfa α (*Tnfα* [45,46,47]), cyclo-oxygenase 2 (*Cox2* [48,49,50,51]), interleukin 6 (*Il6* [46,52]), interleukin 1β (*Il1 β*); and Cd68, the marker of microglia activation [53,54,55]. 

The brain-derived neurotrophic factor (*Bdnf*), reported decreased in serum of PD patients and correlating with motor impairment an DOPAn death [56,57,58], was addressed. 

We analyzed also the risk factors described to be involved in the development of PD: *Snca* (*Park1,4:* α-synuclein) [59,60,61], component of the Lewy-bodies observed in PD and other neurodegenerative pathologies [36,62]. The ubiquitin carboxy-terminal hydrolase L1 (*Uchl1/Park5*), responsible for the clearance of misfolded/aggregated proteins (among them, Snca), and reported as reduced in the cerebrospinal fluid of PD patients [59,63,64,65]. The vesicular monoamine transporter 2 (*Vmat2*) participating in the intracellular vesicular system regulating the cytosolic level of free dopamine, with consequent ROS generation [66,67]. 

Finally apoptosis [30,68], considered an end-point event in the pathology [30], was followed by annexin5 (*Anxa5*).

The release of glutamate in the medium (Glu) was used to monitor glutamate neurotoxicity, leading to excitotoxicity and suggested enhancing DOPAn loss [69,70].

#### 2.3.2. Results at 3 h

As reported in the previous section, at that time the only sign of DOPAn sufferance was the decreased number of neurites, in absence of changes in the number of TH+ DOPAn or neurite length (Figure 2). This experimental time corresponds to a pre-diagnose phase, thus represents the most relevant timing to address in order to individuate the molecular effectors of DOPAn demise.

As shown in Table 1, at 3 h, the very first alteration we noticed involves *Hmox1* (redox marker), *Tnfα* and *Cox2* (inflammation). *Hmox1* is up regulated of about 1.7 fold (*p* < 0.01), returning to basal levels at 12 h. *Tnfα* and *Cox2* experience an increase of about 1.7 fold (*p* < 0.05) and 1.9-fold (*p* < 0.05), reverting rapidly thereafter to control levels (*Tnfα*), or picking (*Cox2*) at about 2.5-fold at 24 h, (*p* < 0.001) before reverting. The results suggest that redox imbalance and inflammation are the early and simultaneous effectors (causes) of DOPAn demise.

#### 2.3.3. Results at 12 h

At 12 h, the morphometric analysis of DOPAn presented a picture comparable with diagnosis (Figure 2). At this experimental point, inflammation and redox imbalances are still present, as revealed by the induction of *Il6* and *Srnx1* (Table 1, 12 h). *Il6* is significantly increased at 12 h (*p* < 0.05), picking at 24 h (about 5.5-fold vs. controls; *p* < 0.001) and then decreasing to control levels. *Sxrn1* up-regulation (1.7-fold, *p* < 0.05 at 12 h) is maintained along the experimental period 48 and 96 h: both *p* < 0.05). The data suggest that, after the first hint, sustained inflammation and oxidative stress push TH+ DOPAn loss. 

#### 2.3.4. Results at 24 h, 48 h and 96 h

As shown in Table 1, at 24 h, *Bdnf* is significantly up-regulated (*p* < 0.001), falling below control levels at 48 and 96 h (both about 0.2 fold, both *p* < 0.01). At 24 h, we notice also a significant glutamate release (*p* < 0.05), continuing all long the 96 h (48 h, and 96 h; both *p* < 0.01).

After 48 h of challenging, apoptosis (*Anxa5*, *p* < 0.05) starts, increasing even more later on (96 h, *p* < 0.001).

Only at the end (96 h), microgliosis (*CD68*, *p* < 0.001) is increased, while *Uchl1* (ubiquitination, *p* < 0.01) and *Il1b* (inflammation, *p* < 0.05) are significantly down-regulated.

All the mentioned events come up with a TH+ DOPAn loss compatible with advanced PD diagnosis, thus representing late/consequential, rather than causative, molecular events in the pathology. 

## 3. Discussion

Currently, PD is considered as a direct consequence of dopaminergic neurons loss, starting from the substantia nigra pars reticularis, and later involving the dopaminergic striato-nigral, and the other three dopaminergic paths form the basal ganglia to the cortex and hypothalamus. Despite the enormous effort made by researchers and clinicians, the huge knowledge of the molecular determinants in DOPAn demise, and the agreed concept that PD is a multifactorial pathology, there is still no clear vision of the sequence of the steps leading to dopamine loss in PD. A model characterized by a slow and progressive degeneration of dopaminergic cells of the SN will, therefore, be useful to allow an assessment of the pre-diagnosis molecular events [21] by a comparison of markers representative of the major molecular effectors of DOPAn demise. 

To the best of our knowledge, only one original study tried to compare the higher number of potential causative mechanisms by a time-course approach [22]. In this study, at the shorter time (3 h) all the investigated genes: inflammation (*Il1β*); oxidative stress (glutathione reductase and glutathione peroxidase precursor), glutamate excitotoxicity (*N*-methyl-d-aspartate 2a receptor); and growth factors (*Gdnf*: glial cell-derived neurotrophic factor) were already altered. The immunohistochemistry also revealed a dramatic (compatible with diagnosis) decrease of TH+ DOPAn density. Altogether, these findings suggest that the experimental scheme applied (50 mg/kg intra peritoneal MPTP: 1-metil 4-fenil 1,2,3,6-tetraidro-piridina to C57Bl mice), was too strong to create a slowly degenerative model, and indicating the need to inducing a more gradual damage, allowing to gain access to the pre-clinical phase and better unravel the temporal sequence of the molecular events in respect to the earliest DOPAn sufferance, before the starting of DOPAn loss. 

**Figure 3:**
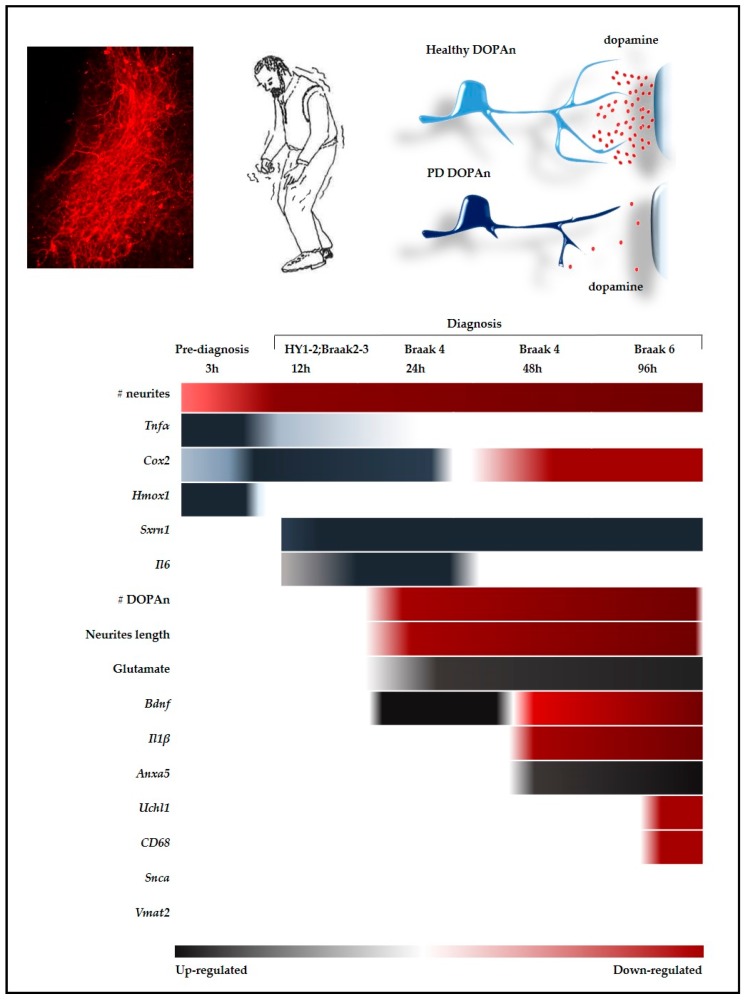
Temporal alterations of the markers evaluated in the study. The picture summarizes the dynamic of all the parameters alterations studied in the work by a time scale approach, tempting a parallel with the clinical situation (for details see text). Red: down-regulated. Black: up-regulated. No color: unchanged. h: hours.

As illustrated by Figure 3, the morphometric data we obtained indicates that we created an ex vivo model of PD with a slow and progressive degeneration of DOPAn, well agreeing with the clinical scenario, and allowing to follow the pathology progression from the pre-diagnosis phase (3 h) to the most advanced stages (24–96 h). 

The most important finding is that we obtained a temporal window (Figure 3, 3 h) corresponding to a pre-diagnosis stage, where the number of DOPAn TH+ cells, as well the length of neurites, is comparable to controls. At that timing the unique sign of DOPAn sufferance is a 27% decrease in the number of neurites, a reduction considered not sufficient to be symptomatic [29]. Therefore, this temporal window (3 h) is crucial for the identification of the molecular events triggering the DOPAn demise (resumed in Figure 3).

At the 3 h time (pre-diagnosis phase), we detected an increased expression of redox stress markers (*Hmox1*) and inflammation markers (*Tnfα*, *Cox2*). Redox imbalance is usually considered the first hint in PD [6,37]. An increase of Hmox1 has been reported in the serum of subjects with a diagnosis of PD [38], and, in autopsy, in the peripheral regions of Lewy bodies [71], where it is supposed to enhance the toxicity by deposition of iron, one of its products [71,72]. By the other side, Hmox1 is not only a sensor for the presence of redox stress but also an inducer of adaptive mechanisms to redox imbalance [73], as well a potential link between redox stress, inflammation and growth factors [74]. 

One of the adaptive mechanisms induced by *Hmox1* is *Srnx1*, always up regulated from 12 h in our model (Figure 3). In models, Srnx1 has been demonstrated to protect from excessive nitrosylation the peroxiredoxins involved in the maintenance of thiol homeostasis [43,75,76], and improving DOPAn survival [42,77], possibly acting on mitochondria [78], another known player in PD. The progressive degeneration of DOPAn in our model, as well the inexorable clinical progression, suggests the failure of the *Srnx1* protective mechanism toward redox imbalance. The *Hmox1* potential protective reaction has been also reported acting through the inhibition of inflammation (down-regulating *Tnfα* and *Il1β*) [74], a feature not observed in our model, nor in the clinic. In fact, an increased level of TNFα has been reported in the serum of PD patients (HY score 2; corresponding to 12 h in our model), and correlating with the cognitive and mood decline [45,46], sleep disorder [45,46,47], as well as correlating with the Hoehn & Yahr progression [45]. The observation again may corroborate the idea of a failure of the adaptive to the damage mechanisms.

Persistent inflammation is a recognized player in PD [30,50,53,79,80], described as contributing to DOPAn demise, and acting as a second hit on the ongoing oxidative imbalance by amplifying it [79,80]. Tacking advantage of our model, allowing accessing the molecular events ongoing the pre-diagnosis stages, we suggest a different scenario, where redox imbalance and inflammation may be simultaneous triggers for DOPAn demise. In fact, in literature, Cox2 is reported as a second-hit pro-inflammatory molecule, supposed to worsen DOPAn degeneration by enhancing both inflammation and redox stress [79,80]. Based on our model, *Cox2* (and inflammation) is a first hit, already significantly up-regulated at 3 h (pre-diagnosis point, together with redox markers), and picking at 24 h (Braak 4). The up-regulation of *Tnfα* at the same earlier (3 h) timing supports this interpretation.

Completing the inflammatory picture, in the model *Il6* is upregulated at 12 h (HY 1/2, Braak 2/3, Figure 3), picking at 24 h (Braak 4). In agreement, Il6 has been described to be increased in the serum of PD subject (HY score 2), and correlating with the worsening of cognition, locomotor speed, and mood (46). Notably, Il6 has been reported enhancing ROS by induction of iNOS (inducible nitric oxide synthase), increasing redox stress and nitrosylation [79,80], thus amplifying the toxic cascade, and possibly explaining the failure in the adaptive-to stress mechanisms. 

Lately on our model (48/96 h, corresponding to Braak 4/6, Figure 3) microgliosis (*CD68*) and apoptosis (*Anxa5*) markers are also increased. Microgliosis is always detected in autopsy of PD brains [48,49,50,53,54,55], and considered crucial to DOPAn loss [6,30]. Alternately, apoptosis has been previously suggested as a final step in PD degeneration [30]. Based on our model, DOPAn TH+ loss (24 h) largely precede the alteration of both microgliosis and apoptosis markers, suggesting that both death mechanisms, and not only apoptosis, are late and consequential events in the dopamine loss.

Among the final events of dopamine demise, we notice also the alteration of the ubiquitination mediated clearance of unfolded proteins (*Uchl1*, 96 h), in line with the clinical scenario [59,63]. Notably, unfolded synuclein (*Snca*) is one of the targets of *Uchl1.* Thus, the temporal windows we described in our model, may agree with the concept that dopamine loss precede synucleinopathy, as previously described [3,36]. 

Of notice, *Uchl1* is a known target for nitrosylation (destroying the enzymatic activity of its targets) occurring in case of excessive oxidative stress [81]. Thus, the reduction of *Uchl1* may be another (together with DOPAn TH+ loss and increased inflammation, see above in the discussion) potential consequence of the inefficient adaptation to stress.

Oxidative stress, and specifically Hmox1 modulation, has been linked also to the induction of growth factors (Gdnf, Bdnf) in animal models of PD [74], with consequent reduction in the DOPAn loss. Bdnf has been consistently reported protecting neurons by an anti-apoptotic effect [82,83,84], by inhibiting microgliosis [11,53], and by inducing the formation of synapses at the dendritic spines [85]. In clinics, BDNF depletion in brain (up to 70% in SN and 80% in DOPAn) has been correlated with DOPAn loss [56], and BDNF decrease in the serum of PD patients has been reported correlating with the timing from diagnosis, pathology severity [58], and cognitive deficit [86,87]. Most importantly, Scalzo [58] noticed an increase of BDNF during the early disease, interpreting this trend as a tentative reaction to DOPAn degeneration. This description fits well with our data (Figure 3), showing an initial significant up-regulation of *Bdnf* (24 h, tentative reaction), followed by a trend to decreasing under the level in controls thereafter (48/96 h). Altogether, the data may support the idea of the failure of the potential adaptive to stress mechanisms. 

We notice also an increase of glutamate release starting from 24 h, when DOPAn neurites loss starts (Figure 3), and persisting to the end of the experimental timing (96 h), suggesting its role in worsening the dopaminergic neurons loss. This may fits with chemically induced rodent models of PD [88], where glutamate neurotoxicity has been proposed to accelerate DOPAn loss by excitotoxicity [69,70]. In the described models, the attenuation of excitotoxicity by the induction of glutamate transporters, improved DOPAn survival, synaptic proteins expression and motor functions [89], while the blockage of induced neurotoxicity by MK801 attenuated the DOPAn loss [69]. Supporting the potential toxic role of glutamate in PD, the administration of drugs able to improve the astrocytes glutamate uptake (responsible for the 90% of extracellular glutamate brain clearance [88]), exhibits a beneficial effects in humans [17,90]. Indeed, extracellular glutamate regulates also immune response and microglia activation [91], supporting one more time the close relationship among all the analyzed molecular players.

The links between the damaging mechanisms reported in literature and fitting with our data, stress two critical aspects of the pathology: 

(1) The importance of blocking the early, inducing steps, to avoid the priming of the self-promoting and self-amplifying cascade of toxic events ongoing DOPAn degeneration, before it became uncontrollable.

(2) Might explain the inefficacy of the current treatment schemes focused in facing a single molecular target. 

For this reason, and based on our results, we suggest a cocktail approach with a wide spectrum anti-inflammatory and an anti-oxidant.

Admittedly, this study lacks of the assessment of the protein(s) content related to the genes variations. However, the selection of the genes we investigated was based on previous data showing a correlation between gene(s) and protein(s) variation [39,40,41,44,51,56,60,61,64,65], rendering the results reliable. 

## 4. Materials and Methods 

### 4.1. Organotypic Brain Culture Preparation 

Organotypic cultures were prepared as described before, with few modifications [92]. Wistar Han TM Rats at 5 days after birth (P5) were obtained from the animal facility of the University of Trieste. Immediately after sacrifice by decapitation, the ventral tegmental area containing substantia nigra (SN) was dissected and maintained in dissection medium (ice-cold Gey’s Balanced Salt Solution—Sigma-Aldrich, St. Louis, MO, USA, plus d-Glucose 10 mg/mL) until use. A McIlwain tissue chopper (Gomshall Surrey, UK) was used to cut transversely 300 μm slices. Healthy slices were selected for structural integrity under stereomicroscope inspection. Slices were then transferred to sterile, semi-porous Millicell-CM inserts (PICM03050, Millipore, Darmstadt, Germany), fed by 1 mL of media and maintained at 37 °C, 5% CO_2_, 95% humidity in a humidified incubator. The media was changed the day after cutting and every two days thereafter. Slices were maintained in culture 10 days before starting treatment. The whole experimental procedure agreed with the Italian Law (Decree 87–848) and European Community Directive (86-606-ECC), and was authorized by the competent OPBA (Organismo Per il Benessere Animale) of the University of Trieste (23 February 2016, and 10 October 2017).

### 4.2. Cultures Medium and Treatment

To create an ex vivo chronic (96 h) model reproducing all the features of human PD (needing dozens of years), slices were challenged with low doses of rotenone. The medium used to culturing the slices was composed of 65% Basal Medium Eagle (Life Technologies Corporation, Grand Island, NY, USA), 10% heat-inactivated Fetal Bovine Serum (Euroclone, Milan, Italy), 25% Hank’s Balanced Salt Solution (Sigma-Aldrich), 1% l-Glutamine (Life Technologies Corporation), 2% Penicillin/Streptomycin (Life Technologies Corporation), and 10 mg/mL d-Glucose (Sigma-Aldrich).

Rotenone (Sigma-Aldrich) was dissolved in DMSO (Sigma-Aldrich) and diluted to the final concentration of 10 nM in complete medium. At each time point, a well was challenged with rotenone, and one well exposed to the same amount of DMSO required dissolving rotenone (controls).

### 4.3. LDH Test

The amount of total extracellular LDH in media, indicative of membrane leakage, was determined using a CytoTox-ONE™ Homogeneous Membrane Integrity Assay (G7891, Promega, Madison, WI, USA), as previously described [92]). The supernatant was collected at each experimental time-point, and the LDH in medium was quantified following the manufacturer’s instruction. The glutamate contained in the OBCs medium was subtracted from the analysis (blank). The signal was read at 560Ex/590Em by an EnSpire Multimode Plate Reader (PerkinElmer, Waltham, MA, USA). 

### 4.4. Immunofluorescent Staining of Dopaminergic Neurons

To follow the morphological degeneration of DOPAn, the cells number, the number of dopaminergic neuron’s neurites, as well as their length were assessed at 3, 12, 24, 48 and 96 h after challenging with low doses of rotenone. Slices were immediately fixed in 4% neutral buffered formalin for 30 min at 4 °C, washed with PBS, and then glycine 0.1 M (Sigma-Aldrich) in PBS to remove residues of paraformaldehyde. After washing, slices were pre-incubated 1 h in 10% normal donkey serum (Sigma-Aldrich), 1% bovine serum albumin (Sigma-Aldrich) and 0.1% Triton-X100 (Sigma-Aldrich) in PBS (blocking solution). Then, slices were incubated three days at 4 °C with primary polyclonal antibody anti-tyrosine hydroxylase (267 ng/mL, AB152, Millipore, Temecula, CA, USA) in an incubation solution containing 1% normal donkey serum (Sigma-Aldrich), 1% bovine serum albumin (Sigma-Aldrich) and 0.1% Triton-X100 (Sigma-Aldrich). After rinsing three times in PBS for 5 min, slices were incubated with labeled donkey anti-rabbit Alexa fluor 546 secondary antibody (1 μg/mL, A10040, Life Technologies, Carlsbad, CA, USA) overnight at 4 °C in incubation solution. Slices were then washed twice with PBS, stained with Hoechst 33258 (2 µg/mL, Sigma-Aldrich), washed with water and mounted (Dako, Agilent, Santa Clara, CA, USA). Fluorescence was detected by fluorescent microscopy Leica DM2000 (Leica Microsystems Srl, Solms, Germany).

### 4.5. Counting of Dopaminergic Neurons and Neurites

Tyrosine hydroxylase-positive (TH+) DOPAn, the number of neurites, and neurites length were counted using a Leica DM2000 fluorescence microscope (Leica Microsystems Srl) by three separate individuals. For DOPAn counting, all the TH positive cells in the 2/3 slices challenged with rotenone (or vehicle) at each experimental timing were counted under a 10× magnification.

Fiji software (ImageJ) [93,94] was used to trace the neurite length of DOPAn, and then expressed in microns. The number of neurites was expressed as the number of neurites for each cell in the treated slices relative to the number of neurites in control. The number of DOPAn in exposed slices was expressed relative to the number of cells in control. DOPAn neurites number and length was assessed (20× magnification) on at least three fields for each slice challenged with rotenone (or vehicle) at each experimental timing, to obtain morphological detail. 

### 4.6. Glutamate Quantification in Culture Media

The amount of extracellular glutamate, a marker of excitotoxicity, was quantified using a Glutamate Assay Kit (MAK004, Sigma-Aldrich). Briefly, after the rotenone exposure, the supernatant was collected, and the assay was performed according to the manufacturer’s instructions. The absorbance (450 nm), proportional to the glutamate released in the medium, was determined using an EnSpire Multimode Plate Reader (PerkinElmer). Glutamate release in media was expressed as fold change compared to the control slices.

### 4.7. Quantitative Real-Time PCR of Selected Marker Genes

Genes reported to be involved in the biomolecular pathways of neuronal degeneration were chosen as markers of these pathways. The mRNA expression of the genes of interest was analyzed by quantitative real-time PCR. Total RNA was extracted using TRI Reagent^®^ RNA Isolation Reagent (Sigma-Aldrich), following the manufacturer’s instructions. Only mRNA samples passing the quality test (230–260–280 nm spectrophotometric validation) were retro-transcribed and used in the study. Complementary DNA (cDNA) was synthesized with the High Capacity cDNA Reverse Transcription Kit (Applied Biosystems, Monza, Italy).

For the quantitative real-time PCR (qPCR), primers were designed using the Beacon Designer 4.2 software (Premier Biosoft International, Palo Alto, CA, USA) on rat sequences available in GenBank (Table 2). The reaction was performed in an iQ5 Bio-Rad thermal cycler (BioRad Laboratories, Hercules, CA, USA). Briefly, 25 ng of cDNA and the corresponding gene-specific sense/antisense primers (250 nM each, except *Cox2* and *Il1β*, 500 and 750 nM, respectively) were diluted in the Sso Advance SYBER green supermix (Bio-Rad Laboratories, Hercules, CA, USA). The genes of interest were analyzed by performing the qPCR as follow: 95 °C for 3 min; a 40 times repetition of: 95 °C for 20 s, 60 °C for 20 s, and 72 °C for 30 s. Finally, the reaction was stopped by an additional step at 72 °C for 5 min. 

At the end of the amplification, we performed a melting curve analysis to confirm the amplification of the desired targets. We never observed non-specific products, primer dimers, or contaminations.

The relative quantification was made using the iCycler iQ software, version 3.1 (Bio-Rad Laboratories, Hercules, CA, USA) by the ΔΔ*C*t method, taking into account the efficiencies of the individual genes and normalizing the results to the housekeeping genes (TATA-binding protein: *Tbp*, Glyceraldehyde 3-phosphate dehydrogenase: *Gapdh*) [95,96].

### 4.8. Statistical Analysis

Data were analyzed with GraphPad Prism version 5.00 for Windows (GraphPad Software, La Jolla, CA, USA). Statistical significance was evaluated by one-way analysis of variance (ANOVA), followed by a Tukey-Kramer multiple comparisons test when *p* value ˂ 0.05. A *p* value < 0.05 was considered as statistically significant. The results are expressed as the mean ± standard deviation (S.D.). Details on the number of independent biological repetitions for each experiment are given in figure and table legends.

## 5. Conclusions

In this work, we describe the development of a slowly degenerative ex-vivo model of Parkinson disease, used to identify the early causative mechanisms of dopaminergic neurons sufferance. Based on our results, we suggest the simultaneous activation of inflammation and oxidative stress as triggers of DOPAn demise, possibly sustained by glutamate neurotoxicity. Thus, we suggest a cocktail approach with a wide spectrum anti-inflammatory, an anti-oxidant, and possibly a glutamate blocker.

## Figures and Tables

**Table 1:** Analysis of selected markers.

Gene	3 h	12 h	24 h	48 h	96 h
Redox imbalance					
*Hmox1*	1.68 ± 0.40 **	1.03 ± 0.16	0.64 ± 0.13	1.07 ± 0.21	1.04 ± 0.46
*Srnx1*	1.21 ± 0.35	1.71 ± 0.57 *	1.48 ± 0.38 **	1.69 ± 0.80 *	1.70 ± 0.39 *
Inflammation					
*Tnfa*	1.68 ± 0.54 *	1.47 ± 0.39	1.26 ± 0.19 *	1.58 ± 0.78	0.89 ± 0.29
*Cox2*	1.89 ± 0.55 *	1.40 ± 0.74	2.43 ± 0.69 ***	0.43 ± 0.58	0.31 ± 0.21
*Il6*	0.82 ± 0.44	2.10 ± 0.60 *	5.52 ± 0.81 ***	0.92 ± 0.68	0.88 ± 0.46
*Cd68*	1.35 ± 0.36	0.93 ± 0.19	0.87 ± 0.15	2.16 ± 0.82	4.20 ± 1.91 ***
*Il1b*	1.58 ± 0.51	1.07 ± 0.37	0.73 ± 0.23	0.83 ± 0.45	0.39 ± 0.12 *
Glutamate neurotoxicity					
Glu	1.12 ± 0.2	1.34 ± 0.12	1.38 ± 0.21 *	1.53 ± 0.37 **	1.52 ± 0.54 **
Neurotrophic growth factor					
*Bdnf*	1.16 ± 0.29	0.84 ± 0.48	2.01 ± 0.68 ***	0.19 ± 0.04 **	0.17 ± 0.17 **
Sinucleopathy					
*Snca*	0.80 ± 0.21	1.38 ± 0.14	1.06 ± 0.27	1.09 ± 0.48	0.90 ± 0.60
Ubiquitination					
*Uchl1*	0.88 ± 0.17	1.08 ± 0.14	1.13 ± 0.24	0.63 ± 0.19	0.46 ± 0.22 **
Vesicular transport					
*Vmat2*	1.11 ± 0.21	1.16 ± 0.56	0.46 ± 0.28	1.07 ± 0.38	0.47 ± 0.11
Apoptosis					
*Anxa5*	1.20 ± 0.14	1.20 ± 0.31	1.00 ± 0.23	1.57 ± 0.37 *	1.79 ± 0.58 ***

mRNA levels and glutamate release in medium in rotenone challenged cultures were expressed as fold vs. DMSO-treated slices. h: hours. *Hmox1*: heme oxygenase1; *Srnx1*: sulfiredoxin 1; *Tnfα*: tumor necrosis factor α; *Il6*: Interleukin 6; *Cox2*: cyclo-oxygenase 2; *Cd68*: cluster of differentiation 68; *Il1β*: interleukin 1β; Glu: glutamate release; *Bdnf*: brain-derived neurotrophic factor; *Snca*: alpha-synuclein; *Uchl1*: ubiquitin carboxy-terminal hydrolase L1; *Vmat2*: vesicular monoamine transporter 2, *Anxa5*: annexin 5. Data are expressed as the mean ± S.D. of at least five independent repetitions. Statistical significance: * *p* < 0.05; ** *p* < 0.01; *** *p* < 0.001.

**Table 2:** Primers used to analyze gene expression of selected genes.

Gene	Accession Number	Forward 5′-3′	Reverse 3′–5′
*Gapdh*	NM_017008.2	CTCTCTGCTCCTCCCTGTTC	CACCGACCTTCACCATCTTG
*Tbp*	NM_001004198.1	CAATGACTCCTATGACCCCT	TTTACAGCCAAGATTCACGG
*Bdnf*	NM_012513.4	GGACATATCCATGACCAGAA	GGCAACAAACCACAACAT
*Uchl1*	NM_017237.3	GGAACTGAAGGGACAAGAAG	ATCCATCCTCAAATTCCAGC
*Vmat2*	NM_013031.1	AACGTCGCCAAATGTTTAAC	CAATGGATGGTGGGACTAAG
*Snca*	NM_019169.2	ACCCCTCTTGCATTGAAATT	CATGAACACATCCATGGCTA
*Hmox1*	NM_012580.2	GGTGATGGCCTCCTTGTA	ATAGACTGGGTTCTGCTTGT
*Srxn1*	NM_001047858.3	AAGGCGGTGACTACTACT	TTGGCAGGAATGGTCTCT
*Tnfα*	NM_012675.2	CAACTACGATGCTCAGAAACAC	AGACAGCCTGATCCACTCC
*Il6*	NM_012589.1	GCCCACCAGGAACGAAAGTC	ATCCTCTGTGAAGTCTCCTCTCC
*IL1β*	NM_031512.2	AACAAGATAGAAGTCAAGA	ATGGTGAAGTCAACTATG
*Cox2*	NM_017232.3	CTTTCAATGTGCAAGACC	TACTGTAGGGTTAATGTCATC
*CD68*	NM_001031638.1	ACTTGGCTCTCTCATTCC	GACTGTACTGTGGCTCTG
*Anxa5*	NM_013132.1	TAATGACCAAAGCTGTCTCG	TTGATTGACAGCACTTCCAA

*Gapdh*: Glyceraldehyde 3-phosphate dehydrogenase; *Tbp*: TATA-binding protein; *Bdnf*: Brain-derived neurotrophic factor; *Uchl1*: Ubiquitin carboxy-terminal hydrolase L1; *Vmat2*: Vesicular monoamine transporter 2; *Snca*: Alpha-synuclein; *Hmox1*: Heme oxygenase1; *Srnx1*: Sulfiredoxin 1; *Tnfα*: Tumor necrosis factor α; *Il6*: Interleukin 6; *Il1β*: Interleukin 1β; *Cox2*: Cyclo-oxygenase 2; *Cd68*: Cluster of differentiation 68; *Anxa5*: Annexin 5.

## Abbreviations

PD	Parkinson disease
DOPAn	Dopaminergic neurons
*SN*	Substantia nigra
*TH+*	Tyrosine hydroxylase
*HY*	Hohen & Yhar score
PET	Positron Emission Tomography
*Hmox1*	Heme-oxygenase 1
*Srnx1*	Sulfiredoxin 1
*ROS*	Reactive oxygen species
*Tnfα*	Tumor necrosis factor alfa α
*Cox2*	Cyclo-oxygenase 2
*Il6*	Interleukine 6
*Il1 β*	Interleukine 1β
*Cd68*	Cluster of differentiation 68, marker of microglia activation
*Bdnf*	Brain-derived neurotrophic factor
*Snca*	Alpha-synuclein (also known as *Park1,4*)
*Uchl1*	Ubiquitin carboxy-terminal hydrolase L1 (also known as *Park5*)
*Vmat2*	Vesicular monoamine transporter 2
*Anxa5*	Annexin5
*Glu*	Glutamate
Gdnf	Glial cell-derived neurotrophic factor

## References

[1] De Lau L.M., Breteler M.M. (2006). Epidemiology of Parkinson’s disease. Lancet Neurol..

[2] Braak H., Braak E. (2000). Pathoanatomy of Parkinson’s disease. J. Neurol..

[3] Shulman J.M., De Jager P.L., Feany M.B. (2011). Parkinson’s disease: Genetics and pathogenesis. Annu. Rev. Pathol..

[4] Dickson D.W. (2012). Parkinson’s disease and parkinsonism: Neuropathology. Cold Spring Harb. Perspect. Med..

[5] Dexter D.T., Jenner P. (2013). Parkinson disease: From pathology to molecular disease mechanisms. Free Radic. Biol. Med..

[6] Hwang O. (2013). Role of oxidative stress in Parkinson’s disease. Exp. Neurobiol..

[7] Antony P.M.A., Diederich N.J., Krüger R., Balling R. (2013). The hallmarks of Parkinson’s disease. FEBS J..

[8] Klemann C.J.H.M., Martens G.J.M., Sharma M., Martens M.B., Isacson O., Gasser T., Visser J.E., Poelmans G. (2017). Integrated molecular landscape of Parkinson’s disease. NPJ Parkinson’s Dis..

[9] Reeve A., Simcox E., Turnbull D. (2014). Ageing and Parkinson’s disease: Why is advancing age the biggest risk factor?. Ageing Res. Rev..

[10] Beal M.F. (1998). Excitotoxicity and nitric oxide in Parkinson’s disease pathogenesis. Ann. Neurol..

[11] Dauer W., Przedborski S. (2003). Parkinson’s disease: Mechanisms and models. Neuron.

[12] Espay A.J., Schwarzschild M.A., Tanner C.M., Fernandez H.H., Simon D.K., Leverenz J.B., Merola A., Chen-Plotkin A., Brundin P., Kauffman M.A. (2017). Biomarker-driven phenotyping in Parkinson’s disease: A translational missing link in disease-modifying clinical trials. Mov. Disord..

[13] Grünblatt E., Mandel S., Jacob-Hirsch J., Zeligson S., Amariglo N., Rechavi G., Li J., Ravid R., Roggendorf W., Riederer P. (2004). Gene expression profiling of parkinsonian substantia nigra pars compacta; alterations in ubiquitin-proteasome, heat shock protein, iron and oxidative stress regulated proteins, cell adhesion/cellular matrix and vesicle trafficking genes. J. Neural Transm..

[14] Lin D., Liang Y., Zheng D., Chen Y., Jing X., Lei M., Zeng Z., Zhou T., Wu X., Peng S. (2018). Novel biomolecular information in rotenone-induced cellular model of Parkinson’s disease. Gene.

[15] Saghazadeh A., Rezaei N. (2015). MicroRNA machinery in Parkinson’s disease: A platform for neurodegenerative diseases. Expert Rev. Neurother..

[16] Youdim M.B.H., Grünblatt E., Levites Y., Maor G., Mandel S. (2002). Early and late molecular events in neurodegeneration and neuroprotection in Parkinson’s disease MPTP model as assessed by cDNA microarray; the role of iron. Neurotox. Res..

[17] Yuan H., Zhang Z.-W., Liang L.-W., Shen Q., Wang X.-D., Ren S.-M., Ma H.-J., Jiao S.-J., Liu P. (2010). Treatment strategies for Parkinson’s disease. Neurosci. Bull..

[18] Zhu M., Li W.-W., Lu C.-Z. (2014). Histone decacetylase inhibitors prevent mitochondrial fragmentation and elicit early neuroprotection against MPP+. CNS Neurosci. Ther..

[19] Freitas M.E., Fox S.H. (2016). Nondopaminergic treatments for Parkinson’s disease: Current and future prospects. Neurodegener. Dis. Manag..

[20] Xu L., Pu J. (2016). Alpha-synuclein in Parkinson’s disease: From pathogenetic dysfunction to potential clinical application. Parkinsons Dis..

[21] Perry V.H. (2012). Innate inflammation in Parkinson’s disease. Cold Spring Harb. Perspect. Med..

[22] Mandel S., Grünblatt E., Maor G., Youdim M.B.H. (2002). Early and late gene changes in MPTP mice model of Parkinson’s disease employing cDNA microarray. Neurochem. Res..

[23] Hawkes C.H., Del Tredici K., Braak H. (2010). A timeline for Parkinson’s disease. Parkinsonism Relat. Disord..

[24] Schapira A.H., Cooper J.M., Dexter D., Jenner P., Clark J.B., Marsden C.D. (1989). Mitochondrial complex I deficiency in Parkinson’s disease. Lancet.

[25] Sherer T.B., Betarbet R., Testa C.M., Seo B.B., Richardson J.R., Kim J.H., Miller G.W., Yagi T., Matsuno-Yagi A., Greenamyre J.T. (2003). Mechanism of toxicity in rotenone models of Parkinson’s disease. J. Neurosci..

[26] Tanner C.M., Kamel F., Ross G.W., Hoppin J.A., Goldman S.M., Korell M., Marras C., Bhudhikanok G.S., Kasten M., Chade A.R. (2011). Rotenone, paraquat, and Parkinson’s disease. Environ. Health Perspect..

[27] Johnosn M.E., Bobrovskaya L. (2015). An update on the rotenone models of Parkinson’s disease: Their ability to reproduce the features of clinical disease and model gene–environment interactions. NeuroToxicology.

[28] Blesa J., Przedborski S. (2014). Parkinson’s disease: Animal models and dopaminergic cell vulnerability. Front. Neuroanat..

[29] Cheng H.-C., Ulane C.M., Burke R.E. (2010). Clinical progression in Parkinson disease and the neurobiology of axons. Ann. Neurol..

[30] Rocha N.P., de Miranda A.S., Teixeira A.L. (2015). Insights into neuroinflammation in Parkinson’s disease: From biomarkers to anti-inflammatory based therapies. BioMed Res. Int..

[31] Hoehn M.M., Yahr M.D. (1967). Parkinsonism: Onset, progression and mortality. Neurology.

[32] Bhidayasiri R., Tarsy D., Bhidayasiri R., Tarsy D. (2012). Parkinson’s disease: Hoehn and Yahr scale. Movement Disorders: A Video Atlas.

[33] Fazio P., Svenningsson P., Cselényi Z., Halldin C., Farde L., Varrone A. (2018). Nigrostriatal dopamine transporter availability in early Parkinson’s disease. Mov. Disord..

[34] Braak H., Del Tredici K., Rüb U., de Vos R.A.I., Jansen Steur E.N.H., Braak E. (2003). Staging of brain pathology related to sporadic Parkinson’s disease. Neurobiol. Aging.

[35] Liu L.-X., Du D., Zheng T., Fang Y., Chen Y.-S., Yi H.-L., He Q.-Y., Gao D.-W., Shi Q.-L. (2017). Detecting dopaminergic neuronal degeneration using diffusion tensor imaging in a rotenone-induced rat model of Parkinson’s disease: Fractional anisotropy and mean diffusivity values. Neural Regen. Res..

[36] Dijkstra A.A., Voorn P., Berendse H.W., Groenewegen H.J., Rozemuller A.J.M., van de Berg W.D.J., Netherlands Brain Bank (2014). Stage-dependent nigral neuronal loss in incidental lewy body and Parkinson’s disease. Mov. Disord..

[37] Blesa J., Trigo-Damas I., Quiroga-Varela A., Jackson-Lewis V.R. (2015). Oxidative stress and Parkinson’s disease. Front. Neuroanat..

[38] Mateo I., Infante J., Sánchez-Juan P., García-Gorostiaga I., Rodríguez-Rodríguez E., Vázquez-Higuera J.L., Berciano J., Combarros O. (2010). Serum heme oxygenase-1 levels are increased in Parkinson’s disease but not in Alzheimer’s disease. Acta Neurol. Scand..

[39] Loboda A., Damulewicz M., Pyza E., Jozkowicz A., Dulak J. (2016). Role of Nrf2/HO-1 system in development, oxidative stress response and diseases: An evolutionarily conserved mechanism. Cell. Mol. Life Sci..

[40] McMahon M., Ding S., Acosta-Jimenez L.P., Frangova T.G., Henderson C.J., Wolf C.R. (2017). Measuring physiological responses to stressors using a novel Hmox1 reporter mouse. bioRxiv.

[41] Chang L.-C., Fan C.-W., Tseng W.-K., Chein H.-P., Hsieh T.-Y., Chen J.-R., Hwang C.-C., Hua C.-C. The Ratio of Hmox1/Nrf2 mRNA Level in the Tumor Tissue Is a Predictor of Distant Metastasis in Colorectal Cancer. https://www.hindawi.com/journals/dm/2016/8143465/abs/.

[42] Li Q., Yu S., Wu J., Zou Y., Zhao Y. (2013). Sulfiredoxin-1 protects PC12 cells against oxidative stress induced by hydrogen peroxide. J. Neurosci. Res..

[43] Zhou Y., Zhou Y., Yu S., Wu J., Chen Y., Zhao Y. (2015). Sulfiredoxin-1 exerts anti-apoptotic and neuroprotective effects against oxidative stress-induced injury in rat cortical astrocytes following exposure to oxygen-glucose deprivation and hydrogen peroxide. Int. J. Mol. Med..

[44] Escobar J., Cubells E., Enomoto M., Quintas G., Kuligowski J., Martinez-Fernandez de la Camara C., Torres-Cuevas I., Sastre J., Belik J., Vento M. (2013). Prolonging in utero-like oxygenation after birth diminishes oxidative stress in the lung and brain of mice pups. Redox Biol..

[45] Kouchaki E., Kakhaki R.D., Tamtaji O.R., Dadgostar E., Behnam M., Nikoueinejad H., Akbari H. (2018). Increased serum levels of TNF-α and decreased serum levels of IL-27 in patients with Parkinson disease and their correlation with disease severity. Clin. Neurol. Neurosurg..

[46] Menza M., Dobkin R.D., Marin H., Mark M.H., Gara M., Bienfait K., Dicke A., Kusnekov A. (2010). The role of inflammatory cytokines in cognition and other non-motor symptoms of Parkinson’s disease. Psychosomatics.

[47] Rocha N.P., Teixeira A.L., Scalzo P.L., Barbosa I.G., de Sousa M.S., Morato I.B., Vieira E.L.M., Christo P.P., Palotás A., Reis H.J. (2014). Plasma levels of soluble tumor necrosis factor receptors are associated with cognitive performance in Parkinson’s disease. Mov. Disord..

[48] Chen H., Jacobs E., Schwarzschild M.A., McCullough M.L., Calle E.E., Thun M.J., Ascherio A. (2005). Nonsteroidal antiinflammatory drug use and the risk for Parkinson’s disease. Ann. Neurol..

[49] Gao X., Chen H., Schwarzschild M.A., Ascherio A. (2011). Use of ibuprofen and risk of Parkinson disease. Neurology.

[50] Bassani T.B., Vital M.A.B.F., Rauh L.K. (2015). Neuroinflammation in the pathophysiology of Parkinson’s disease and therapeutic evidence of anti-inflammatory drugs. Arq. Neuro Psiquiatr..

[51] Teismann P., Tieu K., Choi D.-K., Wu D.-C., Naini A., Hunot S., Vila M., Jackson-Lewis V., Przedborski S. (2003). Cyclooxygenase-2 is instrumental in Parkinson’s disease neurodegeneration. Proc. Natl. Acad. Sci. USA.

[52] Pereira J.R., Santos L.V.D., Santos R.M.S., Campos A.L.F., Pimenta A.L., de Oliveira M.S., Bacheti G.G., Rocha N.P., Teixeira A.L., Christo P.P. (2016). IL-6 serum levels are elevated in Parkinson’s disease patients with fatigue compared to patients without fatigue. J. Neurol. Sci..

[53] More S.V., Kumar H., Kim I.S., Song S.-Y., Choi D.-K. (2013). Cellular and molecular mediators of neuroinflammation in the pathogenesis of Parkinson’s disease. Mediat. Inflamm..

[54] Doorn K.J., Moors T., Drukarch B., van de Berg W.D., Lucassen P.J., van Dam A.-M. (2014). Microglial phenotypes and toll-like receptor 2 in the substantia nigra and hippocampus of incidental lewy body disease cases and Parkinson’s disease patients. Acta Neuropathol. Commun..

[55] Doorn K.J., Goudriaan A., Blits-Huizinga C., Bol J.G.J.M., Rozemuller A.J., Hoogland P.V.J.M., Lucassen P.J., Drukarch B., van de Berg W.D.J., van Dam A.-M. (2014). Increased amoeboid microglial density in the olfactory bulb of Parkinson’s and Alzheimer’s patients. Brain Pathol..

[56] Howells D.W., Porritt M.J., Wong J.Y., Batchelor P.E., Kalnins R., Hughes A.J., Donnan G.A. (2000). Reduced BDNF mRNA expression in the Parkinson’s disease substantia nigra. Exp. Neurol..

[57] Costa A., Peppe A., Carlesimo G.A., Zabberoni S., Scalici F., Caltagirone C., Angelucci F. (2015). Brain-derived neurotrophic factor serum levels correlate with cognitive performance in Parkinson’s disease patients with mild cognitive impairment. Front. Behav. Neurosci..

[58] Scalzo P., Kümmer A., Bretas T.L., Cardoso F., Teixeira A.L. (2010). Serum levels of brain-derived neurotrophic factor correlate with motor impairment in Parkinson’s disease. J. Neurol..

[59] Kumar A., Tamjar J., Waddell A.D., Woodroof H.I., Raimi O.G., Shaw A.M., Peggie M., Muqit M.M., van Aalten D.M. (2017). Structure of PINK1 and mechanisms of Parkinson’s disease-associated mutations. Elife.

[60] Locascio J.J., Eberly S., Liao Z., Liu G., Hoesing A.N., Duong K., Trisini-Lipsanopoulos A., Dhima K., Hung A.Y., Flaherty A.W. (2015). Association between α-synuclein blood transcripts and early, neuroimaging-supported Parkinson’s disease. Brain.

[61] Dumitriu A., Moser C., Hadzi T.C., Williamson S.L., Pacheco C.D., Hendricks A.E., Latourelle J.C., Wilk J.B., DeStefano A.L., Myers R.H. Postmortem Interval Influences α-Synuclein Expression in Parkinson Disease Brain. https://www.hindawi.com/journals/pd/2012/614212/.

[62] Marques O., Outeiro T.F. (2012). Alpha-synuclein: From secretion to dysfunction and death. Cell Death Dis..

[63] McNaught K.S., Jenner P. (2001). Proteasomal function is impaired in substantia nigra in Parkinson’s disease. Neurosci. Lett..

[64] Takami Y., Nakagami H., Morishita R., Katsuya T., Cui T.-X., Ichikawa T., Saito Y., Hayashi H., Kikuchi Y., Nishikawa T. (2007). Ubiquitin carboxyl-terminal hydrolase L1, a novel deubiquitinating enzyme in the vasculature, attenuates NF-κB activation. Arterioscler. Thromb. Vasc. Biol..

[65] Carrieri C., Forrest A.R.R., Santoro C., Persichetti F., Carninci P., Zucchelli S., Gustincich S. (2015). Expression analysis of the long non-coding RNA antisense to Uchl1 (AS Uchl1) during dopaminergic cells’ differentiation in vitro and in neurochemical models of Parkinson’s disease. Front. Cell. Neurosci..

[66] Lohr K.M., Miller G.W. (2014). VMAT2 and Parkinson’s disease: Harnessing the dopamine vesicle. Expert Rev. Neurother..

[67] Osherovich L. (2014). Priming the PD pump. SciBX Sci. Bus. Exch..

[68] Xue G., Hao L.-Q., Ding F.-X., Mei Q., Huang J.-J., Fu C.-G., Yan H.-L., Sun S.-H. (2009). Expression of annexin a5 is associated with higher tumor stage and poor prognosis in colorectal adenocarcinomas. J. Clin. Gastroenterol..

[69] Sonsalla P.K., Albers D.S., Zeevalk G.D. (1998). Role of glutamate in neurodegeneration of dopamine neurons in several animal models of Parkinsonism. Amino Acids.

[70] Morales I., Sabate M., Rodriguez M. (2013). Striatal glutamate induces retrograde excitotoxicity and neuronal degeneration of intralaminar thalamic nuclei: Their potential relevance for Parkinson’s disease. Eur. J. Neurosci..

[71] Schipper H.M., Liberman A., Stopa E.G. (1998). Neural heme oxygenase-1 expression in idiopathic Parkinson’s disease. Exp. Neurol..

[72] Moreau C., Duce J.A., Rascol O., Devedjian J.-C., Berg D., Dexter D., Cabantchik Z.I., Bush A.I., Devos D. (2018). FAIRPARK-II study group iron as a therapeutic target for Parkinson’s disease. Mov. Disord..

[73] Qaisiya M., Coda Zabetta C.D., Bellarosa C., Tiribelli C. (2014). Bilirubin mediated oxidative stress involves antioxidant response activation via Nrf2 pathway. Cell Signal..

[74] Hung S.-Y., Liou H.-C., Kang K.-H., Wu R.-M., Wen C.-C., Fu W.-M. (2008). Overexpression of heme oxygenase-1 protects dopaminergic neurons against 1-Methyl-4-Phenylpyridinium-Induced neurotoxicity. Mol. Pharmacol..

[75] Soriano F.X., Baxter P., Murray L.M., Sporn M.B., Gillingwater T.H., Hardingham G.E. (2009). Transcriptional regulation of the AP-1 and Nrf2 target gene sulfiredoxin. Mol. Cells.

[76] Abbas K., Breton J., Planson A.-G., Bouton C., Bignon J., Seguin C., Riquier S., Toledano M.B., Drapier J.-C. (2011). Nitric oxide activates an Nrf2/sulfiredoxin antioxidant pathway in macrophages. Free Radic. Biol. Med..

[77] Sunico C.R., Sultan A., Nakamura T., Dolatabadi N., Parker J., Shan B., Han X., Yates J.R., Masliah E., Ambasudhan R. (2016). Role of sulfiredoxin as a peroxiredoxin-2 denitrosylase in human iPSC-derived dopaminergic neurons. Proc. Natl. Acad. Sci. USA.

[78] Zhang J., He Z., Guo J., Li Z., Wang X., Yang C., Cui X. (2016). Sulfiredoxin-1 protects against simulated ischaemia/reperfusion injury in cardiomyocyte by inhibiting PI3K/AKT-regulated mitochondrial apoptotic pathways. Biosci. Rep..

[79] Tansey M.G., Goldberg M.S. (2010). Neuroinflammation in Parkinson’s disease: Its role in neuronal death and implications for therapeutic intervention. Neurobiol. Dis..

[80] Tansey M.G., McCoy M.K., Frank-Cannon T.C. (2007). Neuroinflammatory mechanisms in Parkinson’s disease: Potential environmental triggers, pathways, and targets for early therapeutic intervention. Exp. Neurol..

[81] Kumar R., Jangir D.K., Verma G., Shekhar S., Hanpude P., Kumar S., Kumari R., Singh N., Sarovar Bhavesh N., Ranjan Jana N. (2017). S-nitrosylation of UCHL1 induces its structural instability and promotes α-synuclein aggregation. Sci. Rep..

[82] Sullivan A.M., O’Keeffe G.W. (2016). Neurotrophic factor therapy for Parkinson’s disease: Past, present and future. Neural Regen. Res..

[83] Stahl K., Mylonakou M.N., Skare Ø., Amiry-Moghaddam M., Torp R. (2011). Cytoprotective effects of growth factors: BDNF more potent than GDNF in an organotypic culture model of Parkinson’s disease. Brain Res..

[84] Jaumotte J.D., Wyrostek S.L., Zigmond M.J. (2016). Protection of cultured dopamine neurons from MPP(+) requires a combination of neurotrophic factors. Eur. J. Neurosci..

[85] Allen S.J., Watson J.J., Shoemark D.K., Barua N.U., Patel N.K. (2013). GDNF, NGF and BDNF as therapeutic options for neurodegeneration. Pharmacol. Ther..

[86] Khalil H., Alomari M.A., Khabour O.F., Al-Hieshan A., Bajwa J.A. (2016). Relationship of circulatory BDNF with cognitive deficits in people with Parkinson’s disease. J. Neurol. Sci..

[87] Fumagalli F., Racagni G., Riva M.A. (2006). Shedding light into the role of BDNF in the pharmacotherapy of Parkinson’s disease. Pharm. J..

[88] Zhang Y., He X., Meng X., Wu X., Tong H., Zhang X., Qu S. (2017). Regulation of glutamate transporter trafficking by Nedd4-2 in a Parkinson’s disease model. Cell Death Dis..

[89] Zhang Y.-L., Liu Y., Kang X.-P., Dou C.-Y., Zhuo R.-G., Hunag S.-Q., Peng L., Wen L. (2018). Ginsenoside Rb1 confers neuroprotection via promotion of glutamate transporters in a mouse model of Parkinson’s disease. Neuropharmacology.

[90] Sveinbjornsdottir S. (2016). The clinical symptoms of Parkinson’s disease. J. Neurochem..

[91] Rodriguez M., Sabate M., Rodriguez-Sabate C., Morales I. (2013). The role of non-synaptic extracellular glutamate. Brain Res. Bull..

[92] Dal Ben M., Bottin C., Zanconati F., Tiribelli C., Gazzin S. (2017). Evaluation of region selective bilirubin-induced brain damage as a basis for a pharmacological treatment. Sci. Rep..

[93] Schindelin J., Arganda-Carreras I., Frise E., Kaynig V., Longair M., Pietzsch T., Preibisch S., Rueden C., Saalfeld S., Schmid B. (2012). Fiji: An open-source platform for biological-image analysis. Nat. Methods.

[94] Schneider C.A., Rasband W.S., Eliceiri K.W. (2012). NIH image to imageJ: 25 years of image analysis. Nat. Methods.

[95] Vandesompele J., De Preter K., Pattyn F., Poppe B., Van Roy N., De Paepe A., Speleman F. (2002). Accurate normalization of real-time quantitative RT-PCR data by geometric averaging of multiple internal control genes. Genome Biol..

[96] Bustin S.A., Benes V., Garson J.A., Hellemans J., Huggett J., Kubista M., Mueller R., Nolan T., Pfaffl M.W., Shipley G.L. (2009). The MIQE guidelines: Minimum information for publication of quantitative real-time PCR experiments. Clin. Chem..

